# Genotype distribution and allele frequency of thioester-containing protein 1(Tep1) and its effect on development of *Plasmodium* oocyst in populations of *Anopheles arabiensis* in Ethiopia

**DOI:** 10.1371/journal.pone.0311783

**Published:** 2024-10-09

**Authors:** Arega Tsegaye, Assalif Demissew, Ashenafi Abossie, Hallelujah Getachew, Kassahun Habtamu, Teshome Degefa, Xiaoming Wang, Ming-Chieh Lee, Daibin Zhong, James W. Kazura, Guiyun Yan, Delenasaw Yewhalaw

**Affiliations:** 1 Department of Biology, College of Natural Science, Jimma University, Jimma, Ethiopia; 2 Faculty of Health Sciences, School of Medical Laboratory Sciences, Jimma University, Jimma, Ethiopia; 3 Tropical and Infectious Diseases Research Center (TIDRC), Jimma University, Jimma, Ethiopia; 4 Department of Medical Laboratory Sciences, College of Medicine and Health Sciences, Ambo University, Ambo, Ethiopia; 5 Aklilu Lemma Institute of Patho- Biology, Addis Ababa University, Addis Ababa, Ethiopia; 6 Department of Medical Laboratory Sciences, College of Medicine and Health Sciences, Arbaminch University, Arbaminch, Ethiopia; 7 Department of Medical Laboratory Sciences, College of Health Sciences, Arbaminch, Ethiopia; 8 Department of Medical Laboratory Sciences, Menelik II College of Medicine and Health Science, Kotebe University of Education, Addis Ababa, Ethiopia; 9 Department of Microbial, Cellular & Molecular Biology, Addis Ababa University, Addis Ababa, Ethiopia; 10 Program in Public Health, University of California at Irvine, Irvine, CA, United States of America; 11 Center for Global Health & Diseases, School of Medicine, Case Western Reserve University, Cleveland, OH, United States of America; ICMR-National Institute of Malaria Research, INDIA

## Abstract

**Background:**

Thioester-containing protein 1 (TEP1) is a crucial component of mosquitoes’ natural resistance to parasites. To effectively combat malaria, there is a need to better understand how TEP1 polymorphism affects phenotypic traits during infections. Therefore, the purpose of this study was to determine the Tep1 genotype frequency in malaria vector populations from south-western Ethiopia and investigate its effect on *Plasmodium* oocyst development in *Anopheles arabiensis* populations.

**Methods:**

Using standard dippers, *Anopheles* mosquito larvae were collected from aquatic habitats in Asendabo, Arjo Dedessa, and Gambella in 2019 and 2020. Collected larvae were reared to adults and identified morphologically. Female *An*. *gambiae* s.l. were allowed to feed on infected blood containing the same number of gametocytes obtained from *P*. *falciparum* and *P*. *vivax* gametocyte-positive individuals using indirect membrane feeding methods. Polymerase Chain Reaction (PCR) was used to identify *An*. *gambiae* s.l. sibling species. Three hundred thirty *An*. *gambiae* s.l. were genotyped using Restricted Fragment Length Polymorphism (RFLP) PCR and sub samples were sequenced to validate the TEP1 genotyping.

**Results:**

Among the 330 samples genotyped, two TEP1 alleles, TEP1*S1 (82% frequency) and TEP1*R1 (18% frequency), were identified. Three equivalent genotypes, TEP1*S1/S1, TEP1*R1/R1, and TEP1*S1/R1, had mean frequencies of 65.15%, 2.12%, and 32.73%, respectively. The nucleotide diversity was ranging from 0.36554 to 0. 46751 while haplotype diversity ranged from 0.48871 to 0.63161, across all loci. All sample sites had positive Tajima’s D and Fu’s Fs values. There was a significant difference in the TEP1 allele frequency and genotype frequency among mosquito populations (p < 0.05), except populations of *Anopheles arabiensis* from Asendabo and Gambella (p > 0.05). In addition, mosquitoes with the TEP1 *RR genotype were susceptible and produced fewer *Plasmodium* oocysts than mosquitoes with the TEP1 *SR and TEP1 *SS genotypes.

**Conclusion:**

The alleles identified in populations of *An*. *arabiensis* were TEP1*R1 and TEP1*S1. There was no significant variation in TEP1*R1 allele frequency between the high and low transmission areas. Furthermore, *An*. *arabiensis* carrying the TEP1*R1 allele was susceptible to *Plasmodium* infection. Further studies on vector-parasite interactions, particularly on the TEP1 gene, are required for vector control techniques.

## Background

Malaria is the major public health problem. In 2022 alone, 85 malaria-endemic countries reported 608 thousand malaria-related deaths and 249 million malaria cases. In addition, the majority of malaria deaths (94%) and cases (82%) occurred in Africa [1]. Due to the complex *Plasmodium* life cycle, the gametocyte is the only parasite stage that can keep the cycle going in a mosquito. After zygote formation, it develops to okinates, oocysts, and then to sporozoites, the infectious stage to humans [2].

Environmental modification, intensive vector control measures, and climate change might favor malaria vector genotypes or species to adapt to new breeding habitats [3–9]. These factors might lead to changes in vectorial capacity, exert selection pressure, and alter the frequencies of thioester-containing protein 1 (TEP1) alleles. This may lead to the survival and continued transmission of malaria by efficient vectors such as *An*. *arabiensis* and *An*. *funestus*. The susceptibility or refractoriness of a vector to *Plasmodium* parasites defines vector competence and is influenced partially by TEP1, which plays an important role in immunity against pathogens [10–13].

Due to the TEP1 gene’s high polymorphism, different alleles provide varying degrees of refractoriness to infections. The TEP1 gene is located on chromosome 3L. It is a 1338-amino acid-long protein that exhibits genetic polymorphisms linked to different genotypes in its resistance to *Plasmodium* parasite infection [14–16]. It also leads to phenotypic divergence. The TED region of TEP1 contains amino acid sequence variants that can be distinguished from one another.

There are two phenotypes of TEP1: resistant TEP1*R and susceptible TEP1*S. Mosquitoes with the TEP1*R allele is more successful in killing parasites. Because mosquitoes with TEP1*R/*R genotype are totally resistant, whereas TEP1*S/*S mosquitoes are susceptible to infection [17]. Although all *An*. *gambiae* s.l. species have susceptible and resistant alleles, there are notable differences in their geographical distribution and intraspecific variation. Furthermore, TEP1*R alleles affect male fitness in wild *An*. *gambiae* s.l. populations. Moreover, mosquitoes that carrying the TEP1*S alleles exhibit higher rates of fertility due to their ability to eliminate faulty sperm [18].

Furthermore, the frequency and distribution of the TEP1 genotype in *An*. *arabiensis* populations from southern Ethiopia with different malaria transmission settings are not known. Understanding how genetic variation affects mosquito genotypes and how *Plasmodium* adapts in this species is important for effective control of the disease. This information might be used to track infection patterns in vectors, which have a direct effect on the spatio-temporal distribution of malaria. Moreover, information on the impact of the TEP1 gene helps to understand the development of human malaria oocysts in *An*. *arabiensis*, which is a major malaria vector in Ethiopia.

Therefore, it is important to genotype TEP1 in vector populations in order to assess changes that could be responsible for infection rates and potential cases of malaria at various endemicity levels. The genotyping of TEP1 could also help to develop vector control interventions. Understanding the effects of vector control, environmental changes, and underlying biological mechanisms governing vector competence will considerably enhance our understanding of disease transmission dynamics. Thus, this study aimed to determine genotype distribution and frequency of TEP1 and its effect on the development of *Plasmodium* oocyst in populations of *An*. *arabiensis* in malaria-endemic regions of south-western Ethiopia.

## Methods and materials

### Study sites

**Fig 1:**
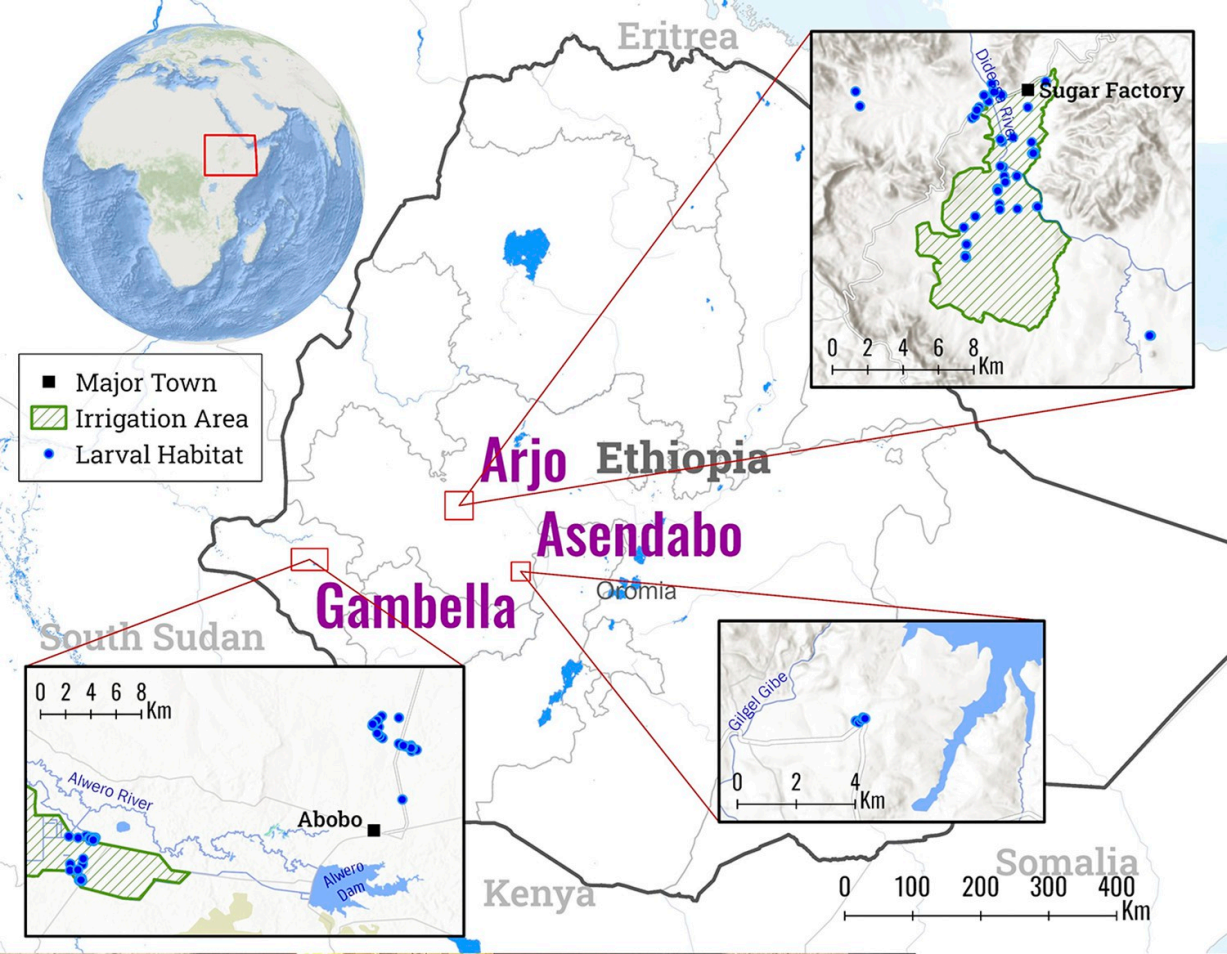
Map of *Anopheles* larval collection sites in south-west Ethiopia.

This study was conducted in three sites in south-western Ethiopia: Arjo Dedessa (00.6029° N, 034.4073° E), Gambella (00.6029° N, 034.4073° E), and Asendabo (00.6029° N, 034.4073° E) (Fig 1). Gambella is an area with high malaria endemicity, whereas Arjo Dedessa is an area with low malaria endemicity and Asendabo is an area with moderate malaria endemicity [19–21]. Asendabo has a large water reservoir for the production of electricity, whereas the other two study regions have areas with environmental changes for irrigated agriculture. Arjo Dedessa and Gambella are areas with irrigated sugarcane plantation and rice farming, respectively.

### Mosquito larval collection and rearing

*Anopheles* larvae were collected using 350-ml standard dippers and hand pipettes. Larvae-breeding habitats were randomly chosen and 20 dips were taken from each habitat in order to decrease the quantity of larvae collected from each breeding habitat. Larvae collected from outside Arjo, particularly Asendabo and Gambella, were transported to the Arjo ICEMR insectary in an aerated container on the same day. *Anopheles* larvae were reared and sorted at the field insectaries. The larvae were reared to adult stages using standardized rearing techniques [22]. A morphological key was used to identify the species. Adults aged 3-5 days were used for membrane feeding assays [23,24].

### Patient recruitment and blood sample collection

Patients visiting Arjo Dedessa sugar factory clinic were diagnosed with *Plasmodium* infections: *P*. *vivax* and *P*. *falciparum* infections by microscopy. Under a microscope, one hundred fields of the blood smear were examined to ascertain the parasite density. The asexual and sexual stages of the parasite were counted against 200 and 500 leukocytes, respectively. The ratio was determined by dividing 8,000 leukocytes per liter of blood by the density of parasites. Of all the positive patients, only those with gametocyte blood stage parasite who consented were enrolled in the study. Patients who refused to give consent and assent, with severe illness, mentally ill, or had taken anti-malarial drugs within the previous two weeks were excluded from the study. All patients who agreed to participate in the study were brought to the International Center for Malaria Research (ICEMR) Laboratory for venous blood sampling with heparin tubes [25]. Venous blood samples (5 ml) were taken from each patient after obtaining informed written consent from patients at the Arjo Didessa Sugar Factory Clinic who were positive for *P*. *vivax* and *P*. *falciparum* infections.

### Artificial mosquito infection experiment

A total of 1152 *An*. *gambiea* s. l. mosquitoes were used in experimental infection using indirect membrane feeding assays to assess the impact of Tep1 on oocyst development in mosquitoes. Using the membrane-feeding apparatus described elsewhere [26], *An*. *gambiea* s. l. mosquitoes were allowed to feed on gametocemic blood preserved in heparin tubes (Vacutainer; BD, Oxford, UK) [26]. Female mosquitoes 3–5 days old were subjected to being starved for 12 hours before feeding on the heparinized blood. Glass feeders with water jackets (mini-feeders; Coelen Glastechniek, Arnemuiden, Netherlands) were attached to a circulating water bath (Julabo GmbH, Seelbach, Germany) maintained at 37°C and covered with a synthetic membrane (parafilm) for feeding. The feeding was performed in the dark room for 30 minutes.

Fully fed mosquitoes were left in their holding cages, while unfed and partially fed mosquitoes were excluded. Until day 7, fully fed mosquitoes were kept in standard laboratory conditions with cotton wool soaked in a 10% sucrose solution. The development of oocysts was examined by dissecting mosquitoes after eight days, and the midgut was stained with 1.0% mercurochrome. Oocyst presence was then examined using a microscope after staining with 1.0% mercurochrome, and oocysts were quantified per mosquito midgut.

### Molecular identification of mosquitoes

Genomic DNA was extracted using the Chelex resin (chelex® -100) method from a randomly selected individual adult female *An*. *gambiae* s.l. following a procedure developed by Musapa *et al*. [27]. 20 μl deionized water were added to individual mosquito sample containing tubes before being crushed into a consistent suspension, and phosphate buffer saline 1X and 10% saponin were gently mixed into the sample homogenates and incubated at room temperature for 20 minutes. The suspension was then centrifuged, and the supernatant was discarded. After being resuspended in 1X PBS, the pellets were gently vortexed, centrifuged, and the supernatant removed. A 20% Chelex-resin solution in deionized water was then added to the pellet suspension in 75 μl of sterile water. The samples were incubated at 85°C for 10 minutes before being centrifuged at 20,000 g for a minute. The DNA was then deposited in prelabeled storage containers. The species-specific polymerase chain reaction (PCR) was used to identify *An*. *gambiae* s.l. as a species [28].

### Genotyping and sequencing

Thioester-containing protein 1 alleles were genotyped using polymerase chain reaction restriction fragment length polymorphism (PCR-RFLP) (29). First, PCR was run using Nest 1 primers (VB3 5′ GATGTGGTGAGCAGAATATGG3′ and VB4 5′ACATCAATTTGCTCCGAGTT3′) targeting 892 base pairs, followed by a second PCR performed on 5 μl of the resulting product from Nest 1 with Nest 2 primers (VB1 5′ ATCTAATCGACAAAGCTACGAATTT3′ and VB2 5′ CTTCAGTTGAACGGTAGTCGTT3′) producing a final fragment length of 758 base pairs. Using Dream Taq Green Master Mix from Thermo Fisher Scientific, the PCR reaction parameters for both samples were set to denaturation at 95°C for 3 min, 35 cycles of 94°C for 30 s, annealing at 55°C for 30 s, extension at 72°C for 30 s, and a final step at 72°C for 6 min. Based on the manufacturer’s instructions, PCR products were digested with the restriction enzymes Bam HI, Hind III, or Bse NI (New England Biolabs Inc), and the results were examined using 2.5% agarose gel electrophoresis. The fragment size of restriction enzyme digestion was subsequently used to categorize the TEP 1 allelic classes. Sequencing 9 corresponding Nested II amplicons from a subset of samples with identified TEP1 alleles was used for confirmation. Using ABI PRISM® 3700 DNA Analyzer and the 3700/3730 BigDye® Terminator v3.1 Sequencing Standard kit, sequencing was performed.

### Data analysis

The genetic diversity indicators, haplotype diversity (Hd), number of haplotypes (h), nucleotide diversity (π of), and mean number of pairwise difference (k)] were calculated using DnaSP Version 6.12.03 [29]. As well as neutrality tests Fu and Li’s D, Tajima’s D, and Fu and Li’s F statistics. The Hardy-Weinberg equilibrium was tested using the Chie square test. MEGA version 11 was used to compare the current study’s sequences to those obtained from GenBank (31). Phylogenetic tree with maximum likelihood 1000 repetitions was built for bootstrapping using

Chie square test χ^2^ = ∑ (O_i_ – E_i_)^2^/E_i_

= ∑ (O_i_ – E_i_)^2^ /E_i +_ (O_i_ – E_i_)^2^/E_i +_ (O_i_ – E_i_)^2^/E_i_

= ∑ (218 – 221.9)^2^ /221.9 _+_ (7– 10.68)^2^/10.68 _+_ (105- – 97.42)^2^/97.42

= ∑ (0.0685) +(1.268) +(0.589)

= 1.925

Df = (3 genotypes) - (2 alleles) = 1 at 95% confidence interval

## Results

### *Anopheles* mosquito species composition

A total of 3,894 Anopheles larvae were collected from diverse breeding sites in Arjo Dedessa, Gambella, and Asendabo, Ethiopia. Of these, 97% successfully developed into adult mosquitoes. The adult population comprised predominantly *Anopheles gambiae* complex (71.8%), followed by *An*. *coustani* complex (14.5%), *An*. *phareonsis* (7.3%), and *An*. *squamous* (3.6%) (Table 1).

**Table 1:** *Anopheline* mosquito species composition in the three malaria endemic settings of southwest Ethiopia; 2019 and 2020.

Study Site	*An*. *gambiae* s.l, n (%)	*An*. *Coustani* complex, n (%)	*An*. *Phareonsis*, n (%)	*An*. *squamous*, n (%)
Arjio Didessa	1152(41.2%%)	275(48.8%)	116(41%)	143(100%)
Gambella	1023(36.6%)	184(32.6)	103(36.4%)	0
Asendabo	620(22.2%)	105(18.6)	64(22.6%)	0
Total	2795(100%)	564(100%)	283(100%)	143(100%)

### Molecular identification of mosquitoes

A total of 386 adults of *An*. *gambiae s*.*l*. were molecularly identified to sibling species using species-specific PCR. Of the 386 molecularly identified *An*. *gambiae* s.l 339 (88.3%) and 37 (9.9%) were *An*. *arabiensis* and *An*. *Amharicus*, *respectively*, while the remaining 19 (4.9%) mosquitoes were not amplified (Table 2). There was significant difference in species abundance among study sites (P < 0.0001) (Table 2).

**Table 2:** Distribution of sibling species of *An*. *gambiae s*.*l*. in different study sites Ethiopia.

Site	*An*. *arabiensis* n (%)	*An*. *amharicus* n (%)
Arjio Deddesa	142(97.9%)	37(23%)
Gambella	114(92.7%)	0
Asendabo	74(92.5%)	0
Total	330(85.5%)	37(9.6%)

Overall, 330 *An*. *arabiensis* samples were genotyped successfully, with three types of genotypes detected. Two TEP1 alleles were identified with frequencies of (82%) TEP1*S and (18%) TEP1*R. With three equivalent genotypes TEP1*SS, TEP1*RR, and TEP1*SR had mean frequencies of 65.15%, 2.12%, and 32.73%, respectively. The highest TEP1*S/S genotype frequency was found in populations of *An*. *arabiensis* from Arjo Didessa (73.9%), which differed significantly from genotype frequency in mosquito populations from Gambella (57.1%), and Asendabo (60.8%) and the difference in genotype frequency among the three populations was significant (p < 0.0001). TEP1*R/R was detected in populations of *An*. *arabiensis* from Arjo Didessa 2 (1.4%) and Gambella 5 (38.6%), but not in populations from Asendabo.

### Hardy Weinberg equilibrium and genetic relationship

Observed Heterozygous was higher than expected Heterozygous and χ2 value 1.925 was significantly less than 3.84 showing that the population deviated from Hardy Weinberg Equilibrium (Table 3).

**Table 3:** Observed and expected allele frequency.

TEP1 genotype	Observed allele frequency	Expected allele frequency	χ2 = $\frac{{\left(Oi-Ei\right)}^{2}}{Ei}$
Homozygous susceptible (S*S)	218	221.9	0.0685
Homozygous Resistance (R*R)	7	10.68	1.268)
Heterozygous (S*R)	105	97.42	0.589
**Total**	330	330	= 1.925

### Genetic diversity indices of TEP1 across mosquito populations from south west Ethiopia

From successfully genotyped *An*. *arabiensis* (n = 330), with samples collected from Arjio (n = 142), Asendabo (n = 74), and Gambella (n = 114) (Fig 1). TEP1 from populations of *An*. *arabiensis* from Arjio and Asendabo demonstrated significant genetic diversity as compared to populations from Gambella (Table 4). The distribution and relative frequencies of the populations are shown in Table 4. Overall haplotype diversity (Hd) and nucleotide diversity (π) were 0.58668 and 0.43244, respectively. In terms of population diversity, haplotype diversity ranged from 0.48871 to 0.63161 and nucleotide diversity ranged from 0.36554 to 0.46751 (Table 5).

**Table 4:** Distribution and frequency of Tep1 genotype in populations of *An*. *arabiensis* from south west Ethiopia.

TEP1 genotype	Arjio population n (%)	Asendabo population n (%)	Gambella population n (%)
Homozygous susceptible (S*S)	105 (73.9)	45 (60.8%)	65 (57.1%)
Homozygous Resistance (R*R)	2 (1.4%)	0	5 (4.3%)
Heterozygous (S*R)	35 (24.7%)	29 (39.2%)	44 (38.6%)
**Total**	142 (100%)	74 (100%)	114 (100%)

**Table 5:** Genetic diversity indices and neutrality tests based on TEP1-TED sequences.

Mosquito population	n	H	Hd	π	K	Fu’s Fs	Tajima’s D	Fu and Li’s D
Arjio	142	3	0.63161	0.46751	460.03	3.4058	2.36410	3.3564
Gambella	114	3	0.52864	0.38296	394.14	3.62485	3.28015	2.81439
Asendabo	74	2	0.48871	0.36554	376.79	4.5704	4.83843	2.8875
Total	330	4	0.58668	0.43244	425.51	3.62840	2.5829	3.70954

### Population structure and differentiation of populations of *An*. *arabiensis* based on TEP1 sequences

There was significant difference in Tep1 gene among populations *An*. *arabiensis* circulating in the three study areas. All the inter-population comparison chi-squares values (Arjio vs Asendabo *x*^2^ = 27.048, df = 3, P = 0.000, Arjio vs Gambella *x*^2^ = 38.292, *df* = 3, P = 0.000, Asendabo vs Gambella *x*^2^ = 3.335, *df* = 2, P = 0.1887) were significant except Asendabo vs Gambella (P > 0.05) (Table 6). Based on pairwise Wright’s fixation index (FST) values, there was significant genetic variation between mosquito populations. Across the three regions, the FST values for *An*. *arabiensis* subpopulations ranged from (-0.01083 –0.07668). (Table 6). The populations of *An*. *arabiensis* from Arjio and Gambella exhibited a low degree of differentiation (FST = 0.07668). Pairwise comparison *FST* values for all populations showed very little genetic differentiation between populations and sites in south-west of Ethiopia. The high level of gene flow (Nm = 12.571) and low Nei’s genetic distance values (0.5) among the populations supported the overall low levels of population structure (FST = 0.019) across all sites.

**Table 6:** Population structure and gene flow among populations of *An*. *arabiensis* from south west Ethiopia.

Mosquito population	χ *2*	df	*P-value*	*F*ST	*Gst*,	*Da*	*Dxy*	*GammaSt*
Arjio vs Asendabo	27.048	3	0.0000	0.05894	0.03758	0.3613	0.45265	0.04057
Arjio vs Gambella	38.292	3	0.0000	0.07668	0.03731	0.03532	0.46055	0.04284
Asendabo vs Gambella	3.335	2	0.1887	-0.01083	0.00364	-0.00337	0.37088	0.00098

### Effects of *TEP1* variation on the development of *Plasmodium* parasite

**Fig 2:**
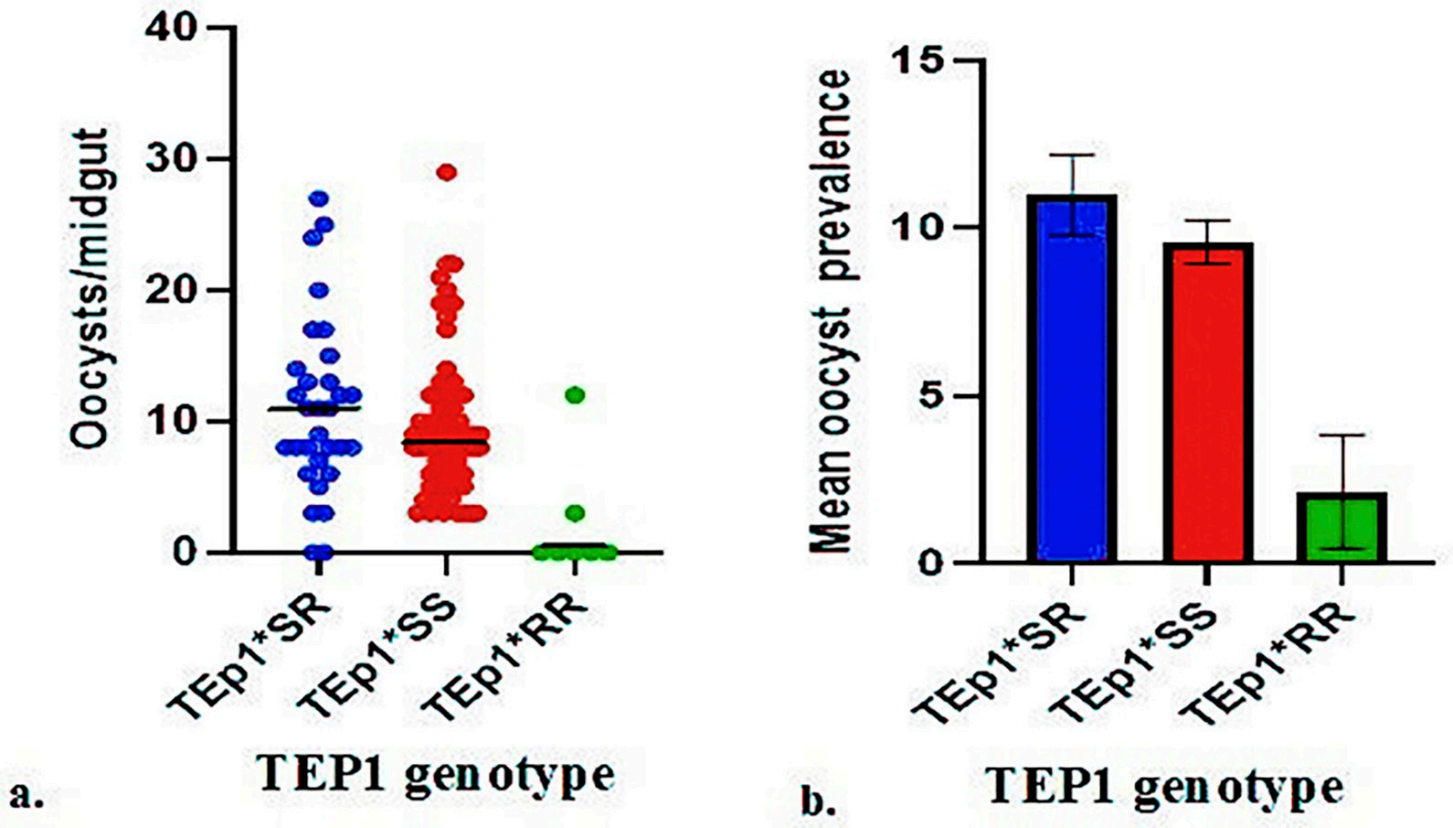
Genotypic differences in *An*. *arabiensis* (a) Oocyst loads per midgut in each Tep1 allele (b) Overall oocyst prevalence in each Tep1 allele.

Experiment results revealed that mosquitos with Tep1*SR genotype had more oocysts per midgut (high oocyst intensity) than mosquitos with genotype Tep1*SS. However, higher infection rate was Tep1*SS than Tep1*SR mosquitos (Fig 2A). The difference in infection prevalence between the *SS and *SR alleles was statistically significant (p = 0.001). The order of increasing mean infection intensity (oocyst burdens) was TEP1*RR< TEP1* SS < TEP1*SR (Fig 1A). Compared to the TEP1 *SR and TEP1 *SS genotypes, the TEP1 *RR genotype was associated with lower *Plasmodium* oocyst formation. There was significant difference in oocyst density between *SR and *SS (p < 0.01). (Fig 2A). TEP1*RR had the lowest infection prevalence (27%), followed by *SS (54%), and *SR (62%), in that order of increasing susceptibility.

### Phylogeny of TEP1 genes in mosquito populations from south-west Ethiopia

**Fig 3:**
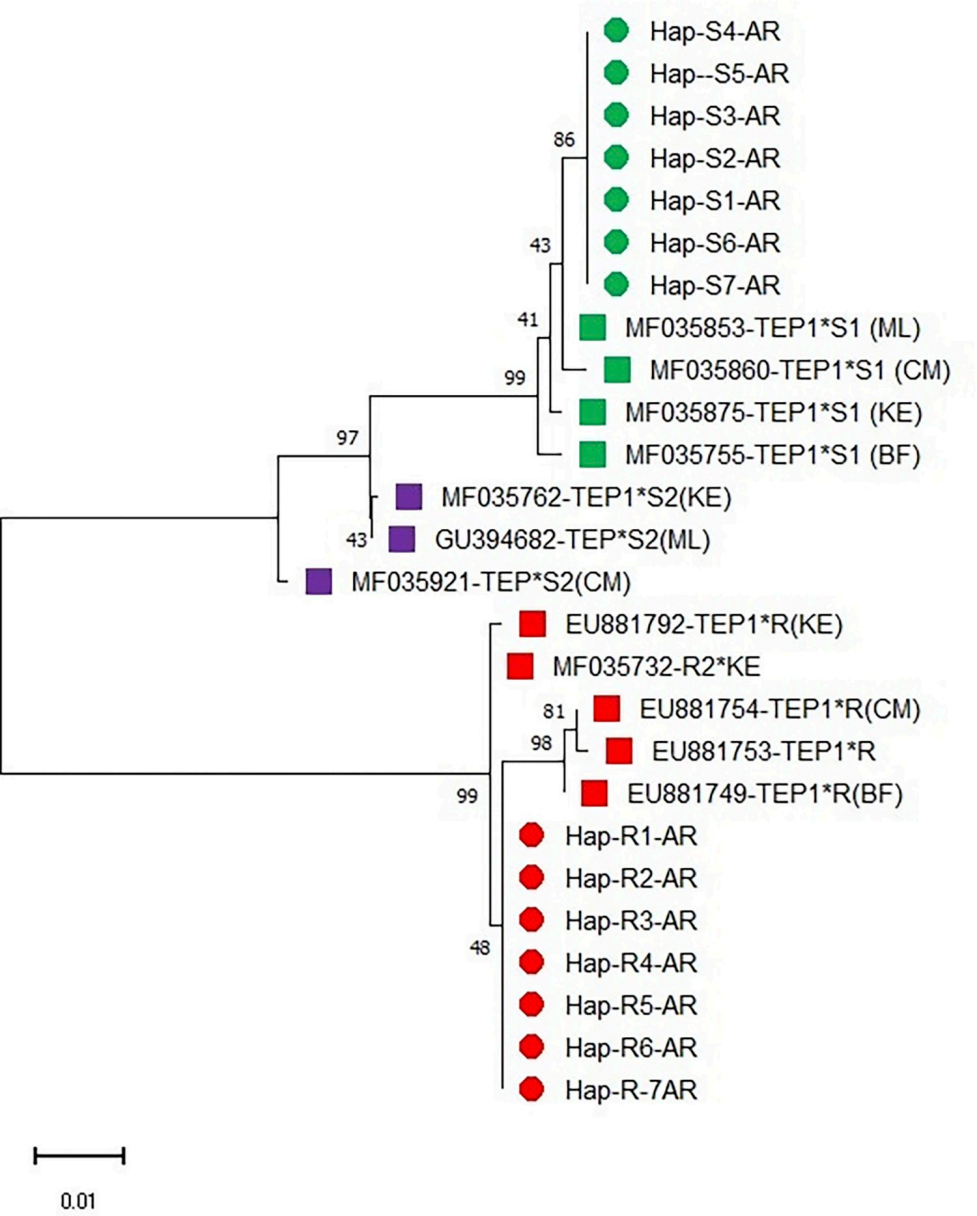
The study used the Maximum Likelihood method [1] and the Kimura 2-parameter model to infer ancestral states [2]. The initial tree was inferred using a pre-computed tree file, and the rates were treated as a Gamma distribution. The final dataset included 710 positions, with evolutionary analyses conducted in MEGA11[3].

There are two major clades: susceptible (TEP1*S) and resistant (TEP1*R) genotypes, which have evolved independently and apart from one another (Fig 3). TEP1*SS alleles found in this study were related to TEP1*S1: MF035875 from Malindi/Busia/Teso Kenya, MF035755 from Vallée de Kou VK5/Somoussou in Burkina Faso, MF035853 Nankilabougou in Mali, and MF035860 Mvan,Mfou in Cameroon. TEP1*S1 evolved as a result of a mutation on the mosquito strain G3 with TEP1*S3 (FN431782), which shared an ancestor with strain 4Arr, which had the TEP1*S2 (MF035921 *S2) allele. TEP1*R1 from the current study shared a common lineage with TEP1*R1 (MF035727*R1), which shared a susceptible ancestral lineage with (S) alleles (Fig 3). The TEP1*S1 allele was more diverse than TEP1*S2 and had a higher prevalence overall. There were three subclades of mosquito populations carrying the resistant allele (TEP1*R1–TEP1*R2 and TEP1*R3). TEP1*RR alleles identified in the study sites shared a common lineage with TEP1*R1 (MF035727) from Mali, Burkina Faso, Cameroon, and Kenya.

## Discussion

In accordance with previous studies in the same study country, the *An*. *gambiae* complex was predominant among the *Anopheles* species [30–33]. Of this complex, the principal malaria vector, *An*. *arabiensis*, makes up 88.3%. This complex comprises the main malaria vector in Africa, and its susceptibility to *Plasmodium* infection varies widely in different malaria-endemic settings [34,35]. Immune gene (TEP1) variations in *An*. *gambiae* s.l. were found and connected to natural refractoriness. The functional impact of TEP1 polymorphism on phenotypic features connected to malaria infections in *An*. *gambiae* s.l. should be determined in order to develop efficient malaria vector control techniques [36–39].

Studies show that there were six allelic classes in Tep1: TEP1*R1, TEP1*R2, TEP1*R3 TEP1*S1, TEP1*S2 and TEP1*S3, have been described in African populations of *An*. *gambiae* complex [17,36,38]. TEP1*R1 and TEP1*S1 alleles were detected in populations of *An*. *arabiensis* from south-west Ethiopia in this study. A higher similarity index was documented between the sequenced alleles detected by RFLP-PCR in this study and the sequences obtained from NCBI. Similar studies in a neighboring country with different malaria transmission settings show that TEP1*S1and TEP1*R2 were the most commonly detected alleles [36,39] circulating in western Kenya. In this study, Tep1*R1 was found in populations of *An*. *arabiensis* from Gambella and Arjio, south-west Ethiopia. However, in Mali, Burkina Faso, Cameroon, and Kenya, Tep1*R1 alleles were found in A. coluzzii [40].

Mosquitoes with thioester-containing protein 1 *S1/S1 had a higher proportion of infection in this study. A similar study in west Kenya found that TEP1*S1/S1 was the most common allele (66%) and the only allele positive for sporozoite infection [41]. Even though a study shows that Tep1*S1 and Tep1*S2 have equal susceptibility. Whereas, in this study, oocyst intensity was much higher in mosquitoes with Tep1*S2 than mosquitoes with Tep1*S1. Despite the fact that TEP1*R1/R1 genotype was totally resistant to *Plasmodium* infection in different studies., However, in this study, some of TEP1*R1/R1 genotype exhibited the lowest infection prevalence (27%). This might be related to P47 genetic diversity; studies indicate that some African parasites, such as the NF54 strain, have developed ways to evade TEP1-mediated immune responses [42,43].

Further studies suggest variation in P47 genetic diversity indices per location was associated malaria transmission intensity [44,45]. The majority of the detected shifts occurred inside the immunogenic domain of the P47 antigen, which is in line with earlier findings [46]. Variations in the P47 antigen have been found to be essential for enabling the parasite to escape nitration or TEP1-mediated death [44]. Despite the fact that NF54 wild strains have a survival rate of over 90% in *An*. *gambiae* R strain mosquitoes, this corresponds to the sequence lacking the mutation [46,47].

Pairwise comparisons for all populations show low FST values for the genetic difference between species and sites in the south-west of Ethiopia. The observed substantial gene flow in Anopheles mosquito populations could be attributed to increased migration facilitated by human transportation networks [48]. Unlike other organisms where geographical barriers such as flight ranges and breeding grounds significantly impede gene flow [49], these factors may be less influential for *Anopheles* mosquitoes in the context of modern transportation systems. While geographical features like mountains, rivers, and valleys can act as barriers to mosquito dispersal and potentially reduce gene flow [50], the ability of certain *Anopheles* species to undertake long-distance flights suggests that they may be capable of overcoming some of these challenges.

A low degree of genetic variation suggests a high degree of gene flow among the study sites, as shown by an effective migration index. Expected heterozygosity values were less than the observed heterozygosity among mosquito populations, suggesting the presence of advantageous alleles [51,52]. This could happen due to the relative high mutation rates and gene flow within populations [53,54]. The deviations from HWE suggest that the TEP1 locus is subject to strong selection as well as other mechanisms such as mutations [55,56] and gene flow, which may influence TEP1 alleles in populations of *An*. *arabiensis* in southwest Ethiopia.

Findings show that low TEP1*R allele frequencies may be the result of selective pressures on the TEP1 gene due to functional variants that enhance *Plasmodium*-infected mosquitoes [57–59]. As a result of these processes that promote evolution at the TEP1 loci [60], and other predisposing factors, these areas continue to have a relatively high malaria prevalence. In addition, population structure is influenced by vector control treatments such as insecticide-treated nets, indoor residual spraying, and environmental factors. Furthermore, the existence of a negative selection indication on this gene suggests that selective purification works on genetic structure by removing harmful mutations [61].

Thioester-containing protein 1 allele clustering in particular vector species may be explained by the similar composition of vector species in different settings and the ongoing selective pressure on this gene. According to earlier research, insecticide resistance, which is currently more prevalent in vectors in high-transmission environments, is blame for the variation in transmission [62–66]. Other studies also presented that comparatively increased quantity of highly effective vectors in the high transmission environment could be human mobility and vector behavior [67,68], as well as environmental factors [69,70]. It also necessitates more research to determine the implications of using the TEP1 gene as a vector control strategy in different situations.

Low TEP1 allele variation was found in vector populations from high and low transmission settings in this study. A similar study carried out in western Kenya [71] found no significant variations in TEP1 alleles in vector populations in different transmission settings. Findings show that significant extended vector control efforts in different malaria-endemic areas may have altered the genetic composition of the vector population, as reported in Dielmo, Senegal, and Western Kenya [72–74].

## Conclusion

This study revealed genetic variations and a low population structure in mosquito populations from high and low malaria transmission areas of south-west Ethiopia. TEP1*R1 and TEP1*S1 were the most common alleles observed in populations of *An*. *arabiensis*. There was no significant difference in TEP1* genotypic frequency between mosquito populations from high and low malaria transmission settings. Furthermore, the population of *An*. *arabiensis* carrying the TEP1*R gene was susceptible to *Plasmodium* infection. Further studies on host-parasite interactions are required to consider the TEP1*R allele for future novel vector control interventions.

## Supporting information

S1 File (XLSX)
